# Consumption of benthic cyanobacterial mats on a Caribbean coral reef

**DOI:** 10.1038/s41598-019-49126-9

**Published:** 2019-09-03

**Authors:** Ethan C. Cissell, Joshua C. Manning, Sophie J. McCoy

**Affiliations:** Department of Biological Science, Florida State University, Tallahassee, Florida USA

**Keywords:** Behavioural ecology, Community ecology

## Abstract

Herbivory is an important process in the general structuring of coral reef benthic communities. However, evidence of its ability to control coral reef benthic cyanobacterial mats, which have recently proliferated on reefs worldwide, remains ambivalent. Here, we report that the French Angelfish (*Pomacanthus paru*), Striped Parrotfish (*Scarus iseri*), Rock Beauty (*Holacanthus tricolor*), Ocean Surgeonfish (*Acanthurus bahianus*), Blue Parrotfish (*Scarus coeruleus*), and Atlantic Blue Tang (*Acanthurus coeruleus*) consume benthic cyanobacterial mats on coral reefs in Bonaire, Netherlands. We documented the foraging patterns of *P*. *paru* and *S*. *iseri*, and found that benthic cyanobacterial mats comprised 36.7% ± 5.8% and 15.0% ± 1.53% (mean ± standard error) of the total bites taken by *P*. *paru* and *S*. *iseri* respectively. This magnitude of consumption suggests that grazing by reef fishes may represent a potentially important, but previously undocumented, top-down control on benthic cyanobacterial mats on Caribbean reefs.

## Introduction

Biodiversity of Caribbean coral reefs has decreased substantially following coral losses over the past half century, threatening overall reef function^1–5^. Identifying and understanding processes that affect reef biodiversity and ecosystem function are therefore pivotal for effective reef management. Coral reef degradation can follow various trajectories, with differing implications for community structure and function, and the stressors that maintain degradation^6^. While increase in macroalgae is often identified as a key indicator of degradation^7^, degraded reefs additionally, or alternatively, can undergo dramatic increases in microbial biomass^8^. Though cyanobacteria, in other forms, are ubiquitous on coral reefs^9^, benthic cyanobacterial mats have historically occurred in low abundances (<1% cover)^10^ on all coral reefs, forming integral components of reef processes such as structure-building, primary productivity, and nitrogen cycling^9,11–13^. However, numerous recent reports document an increase in benthic cyanobacterial mat cover on coral reefs worldwide^8,10,14,15^.

Decadal-scale surveys reveal that benthic cyanobacterial mat cover increased from 0.1% to 22.2% of the substrata on Caribbean coral reefs from 1973–2013, while coral, crustose coralline algal, and even macroalgal cover have decreased^10^. Cyanobacteria can tolerate and benefit from increased water temperature, solar radiation, and nutrient levels which threaten the health and species richness of coral and other susceptible taxa on reefs^16^. Organic matter enrichment, and its subsequent degradation, may be a dominant driver of benthic cyanobacterial mat proliferation^17^. Proliferation of cyanobacterial mats can cause coral disease^18–20^, inhibit coral larval recruitment^21^, cause fish mortality^22^, and smother other benthic organisms by creating anoxic boundary layers^17,23^. Additionally, the high levels of nitrogen fixation associated with benthic cyanobacterial mats^11^ and their net release of dissolved organic carbon^23^ could amplify macroalgal cover on coral reefs, promote pathogenic bacterial expansion, and generate feedbacks that maintain reefs in degraded states^24^.

Herbivory plays an important role in structuring benthic community composition on coral reefs^7,25,26^. However, the role of top-down control by grazing in regulating the abundance and distribution of coral reef benthic cyanobacterial mats remains equivocal. Cyanobacteria, in general, are known to be targeted and consumed by numerous species of both freshwater^27,28^ and marine fishes^29–33^, and can be important nutritional sources for consumers^29,34^. Feeding on mat-forming cyanobacteria, however, has rarely been documented. Some mat-forming cyanobacteria (mostly belonging to the genus *Lyngbya* [or *Dapis*]^35^) produce toxins that are shown to deter grazing by large generalist herbivores (especially reef fishes) in experimental preference assays^22,36–42^, driving the paradigm that mat-forming cyanobacteria are unpalatable to herbivorous fishes. These deterrent effects, however, can vary with the availability of other preferred foods, experience with the toxin-producing cyanobacteria, and with the hunger level of the consumer^42^. Additionally, toxicity can be highly spatially and temporally variable within blooms of cyanobacteria^43,44^. Only one species of large-bodied reef fish in the Pacific, *Bolbometopon muricatum* (Green Humphead Parrotfish), has been previously observed grazing on mat-forming cyanobacteria^29^; no large-bodied herbivores have been documented to consume cyanobacterial mats on Caribbean coral reefs. Small invertebrates (e.g. Opisthobranch molluscs, or ‘sea slugs’) are specialist consumers of mat-forming cyanobacteria^45,46^, but likely have little impact on reef-scale cyanobacterial mat abundance and distribution^8^. Considering the rapid pace of reef cyanobacterial mat expansion, especially on Caribbean reefs^10^, a better understanding of interactions between large-bodied reef grazers and benthic cyanobacterial mats is vital to understand potential mechanisms controlling their proliferation and the future of their population dynamics.

**Figure 1:**
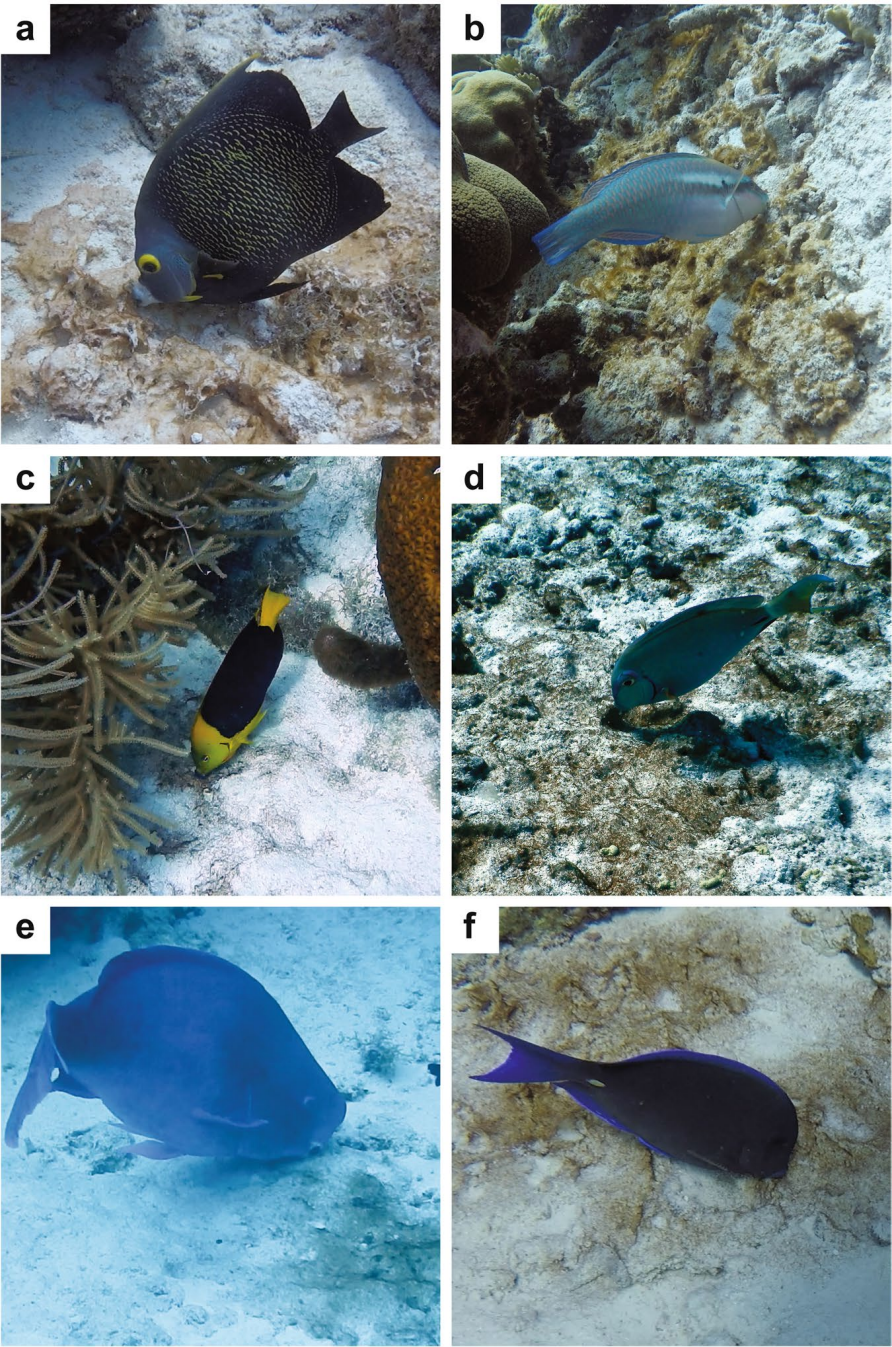
Reef fishes observed grazing on benthic cyanobacterial mats in Bonaire. (**a**) French Angelfish (*P*. *paru*). (**b**) Striped Parrotfish (*S*. *iseri*). (**c**) Rock Beauty (*H*. *tricolor*). (**d**) Ocean Surgeonfish (*A*. *bahianus*). (**e**) Blue Parrotfish (*S*. *coeruleus*). (**f**) Atlantic Blue Tang (*A*. *coeruleus*).

We observed the following 6 different reef fishes grazing on benthic cyanobacterial mats on the fringing coral reefs off the island of Bonaire, Netherlands: French Angelfish (*Pomacanthus paru*), Striped Parrotfish (*Scarus iseri*), Rock Beauty (*Holacanthus tricolor*), Ocean Surgeonfish (*Acanthurus bahianus*), Blue Parrotfish (*Scarus coeruleus*), and Atlantic Blue Tang (*Acanthurus coeruleus*; Fig. 1). We focused on two of these, *P*. *paru* and *S*. *iseri*, for further detailed characterization of feeding behavior. These two species were targeted due to their large size and ubiquity on the reef relative to the other 4 species we observed, leading us to predict they would have the largest impact on reef-scale abundance and distribution of benthic cyanobacterial mats out of the 6 species we observed grazing them. Individual fish (n = 16 *P*. *paru* individuals; n = 13 *S*. *iseri* individuals) were followed for 11 minutes each (Supplementary Table S1) to document their foraging preferences and determine the extent to which these fishes are incorporating benthic cyanobacterial mats into their overall foraging. We quantified the total number of bites taken and the proportions of different benthic feeding substrata bitten by each fish.

## Results

### P.paru

**Figure 2:**
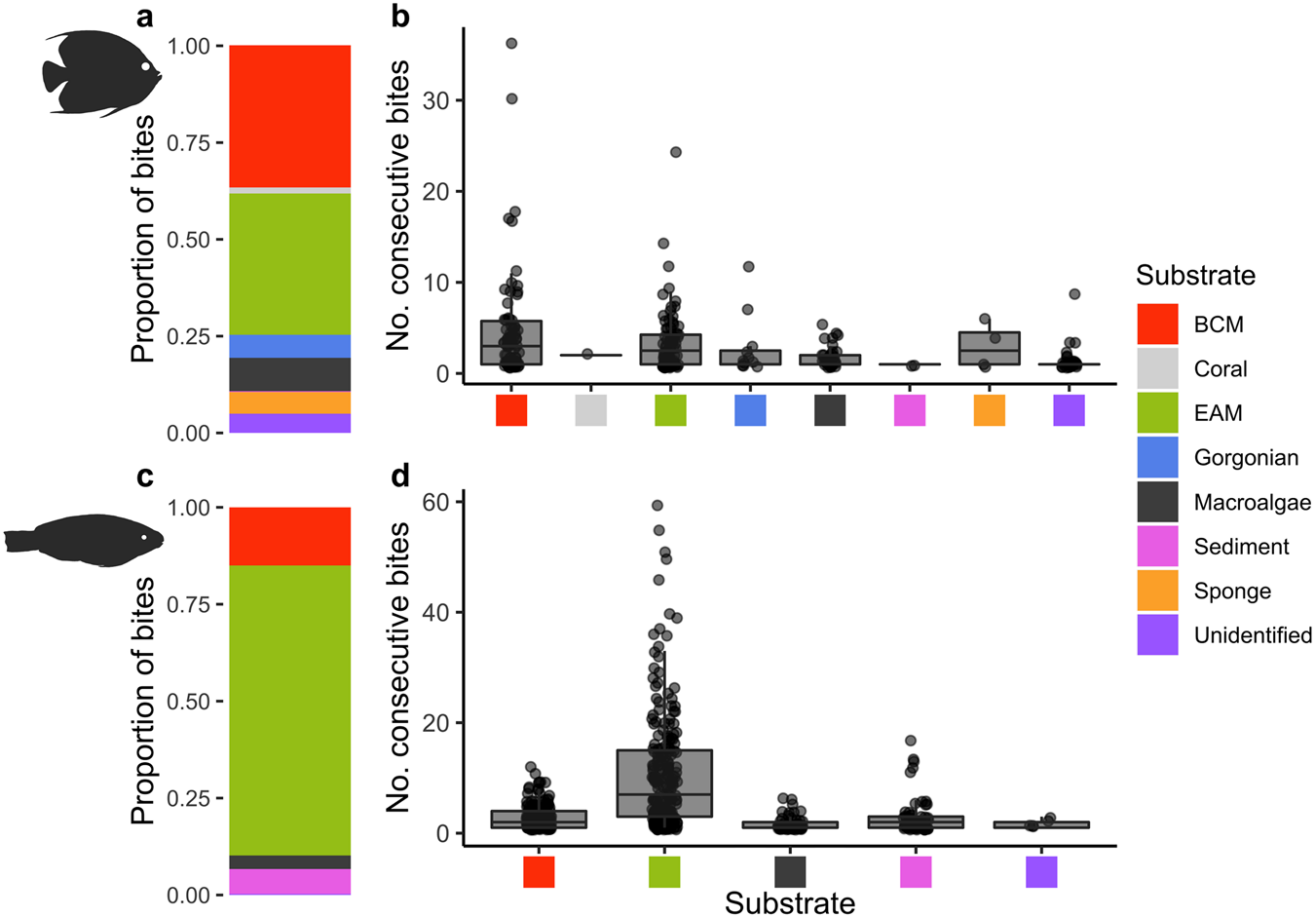
Composition of bites and consecutive bites per substrate for *P*. *paru* and *S*. *iseri*. (**a**) Proportion of total bites for each substrate for *P*. *paru* (mean ± SE): BCM (benthic cyanobacterial mat) 36.7% ± 5.8%; Coral 1.56% ± 1.56%; EAM (epilithic algal matrix) 36.5% ± 4.67%; Gorgonian 5.93% ± 2.73%; Fleshy Macroalgae 8.61% ± 1.93%; Sediment 0.24% ± 0.17%; Sponge 5.57% ± 4.67%; Unidentified 5.0% ± 1.52%. (**b**) Boxplots showing median and spread of consecutive bites for each substrate taken by *P*. *paru* individuals. Circles denote individual counts. (**c**) Proportion of total bites for each substrate for *S*. *iseri* (mean ± SE): BCM 15.0% ± 1.53%; EAM 74.8% ± 3.01%; Fleshy Macroalgae 3.47% ± 0.75%; Sediment 6.44% ± 1.65%; Unidentified 0.28% ± 0.15%. (**d**) Boxplots showing median and spread of consecutive bites for each substrate taken by *S*. *iseri* individuals. Circles denote individual counts.

*P*. *paru* primarily targeted both benthic cyanobacterial mats and epilithic algal matrix (EAM; defined in methods), with sponges, gorgonians, fleshy macroalgae (largely *Dictyota* spp.; hereafter macroalgae), and sediment all representing minor components of the total bites taken across all substrata (Fig. 2a). Benthic cyanobacterial mats comprised 36.7% ± 5.8% (mean ± standard error; SE) of the total bites by *P*. *paru* (Supplementary Table S2). The mean proportion of bites on benthic cyanobacterial mats did not significantly differ from the mean proportion of bites on EAM, 36.5% ± 4.67% (mean ± SE; one sample t-test; t = 0.0415, df = 15, p = 0.968). To determine whether *P*. *paru* was only sampling benthic cyanobacterial mats (i.e. small number of consecutive bites) vs. feeding on benthic cyanobacterial mats, we counted the number of consecutive bites for each fish for each substrate. Though most fish took 0–5 consecutive bites on all substrata, the highest number of consecutive bites, 36, was taken on benthic cyanobacterial mats. Of all instances where a *P*. *paru* individual took more than 5 consecutive bites on the same substrate (n = 36), 18 were on cyanobacterial mats and 14 on EAM, compared with 0–2 on all other substrata (Fig. 2b; Supplementary Table S3).

### S. iseri

The most commonly bitten substrate by *S*. *iseri* individuals was EAM, with benthic cyanobacterial mats, macroalgae, and sediment comprising the remainder of observed bites (Fig. 2c). Benthic cyanobacterial mats comprised 15.0% ± 1.53% (mean ± SE) of their total bites (Supplementary Table S2). The mean proportion of bites on benthic cyanobacterial mats differed significantly from the mean proportion of bites on EAM 74.8% ± 3.01% (mean ± SE; one-sample t test; t = −40.0, df = 12, p < 0.001). Counts of bouts exceeding 5 consecutive bites (n = 151) revealed that *S*. *iseri* individuals consistently took the most consecutive bites on EAM (122 bouts) compared to benthic cyanobacterial mats (20 bouts) and other groups (0–7 bouts; Fig. 2d, Supplementary Table S3).

## Discussion

Cyanobacteria, in general, are known to be targeted by fishes in both marine^29–33^ and freshwater^27,28^ systems as important nutritional sources^29,34^. This includes other members of the genus *Acanthurus*, specifically on coral reefs in the Indian Ocean and Red Sea^31,32^. Thus, it might be expected that the proliferating cyanobacterial mats on coral reefs would likewise be targeted for consumption. However, some toxic mat-forming species are known to deter grazing^22,36–42^, and consequently, benthic cyanobacterial mats are currently viewed as largely unpalatable to large-bodied herbivores. While Clements *et al*. (2016) presents recent evidence that *B*. *muricatum* individuals consume benthic cyanobacterial mats on Pacific reefs^29^, no large-bodied consumers have been documented consuming cyanobacterial mats on Caribbean coral reefs where cyanobacterial mat abundance is rapidly increasing^10^. Here, we observed 6 different species biting benthic cyanobacterial mats (Fig. 1) and documented significant bites on benthic cyanobacterial mats by both *P*. *paru* and *S*. *iseri* (Fig. 2).

In *P*. *paru*, we observed no difference in the proportion of bites on benthic cyanobacterial mats from that on EAM, which suggests equal preference of this species for both substrata. However, calculated selectivity indices suggest that *P*. *paru* selected for benthic cyanobacterial mats, and either neutrally fed on, or avoided, EAM (Supplementary Fig. S1). Further, the high number of consecutive bites observed on benthic cyanobacterial mats by *P*. *paru* individuals (Supplementary Table S3) and the significant positive correlation between total bites taken and bites on BCM (Supplementary Fig. S2) suggests that these individuals were not merely sampling benthic cyanobacterial mats but targeted them as part of their normal foraging behavior. *P*. *paru* individuals occasionally hovered over a single benthic cyanobacterial mat and fed from this same mat for extended durations (Supplementary Video S1). This differed from the feeding behavior we observed with *S*. *iseri*. Extended feeding forays that are typical to this genus^47^ were usually not observed on benthic cyanobacterial mats (Supplementary Video S2), and the number of consecutive bites on these mats were often minimal when compared to feeding on EAM (Fig. 2). However, selectivity indices suggest that, overall, *S*. *iseri* may be selectively sampling benthic cyanobacterial mats (Supplementary Fig. S1).

Cyanobacteria are present in EAM assemblages^29,48^ and as epiphytes on fleshy macroalgae^49^, and can be selected for as the preferred feeding targets as observed in reef parrotfishes^29^. The growing conspicuity of benthic cyanobacterial mats, especially on Caribbean reefs^10^, could lead to greater encounter rates by mobile reef consumers, increasing the likelihood of mat growth forms being selected for and consumed in addition to these other cyanobacterial growth forms. Some animals are known to alter the frequency with which they target different resources, as well as the identity of dominant biting targets, in response to changing resource availability^50^. Likewise, reef fishes could increasingly target and consume benthic cyanobacterial mats in response to their growing abundance.

Other documented behaviors of *P*. *paru* may help explain their ability to consume benthic cyanobacterial mats in such large magnitude. *P*. *paru* may target foods by color^51^, which may have facilitated their incorporation of benthic cyanobacterial mats into their foraging behavior. *P*. *paru* feed on other chemically defended species (i.e. sponges)^51,52^, which might explain their ability to consume often toxin-rich benthic cyanobacterial mats^42^. In this study, we did not document toxin production from the mats we observed being grazed in Bonaire, and thus cannot definitively remark on how the presence or absence of deterring secondary metabolites influenced our behavioral observations. Further work should focus on understanding toxin production in these mats, and how they influence the foraging decisions of these reef grazers.

Since benthic cyanobacterial mats create anoxic boundary layers and promote sediment hypoxia^17,23^, mats may encourage the emergence of sediment infauna^53^ that could be targeted by omnivorous fish such as *P*. *paru*. This is potentially supported by our observation of extensive feeding on mats found on sediment (Supplementary Video S1). However, we also observed *P*. *paru* individuals deftly removing cyanobacterial mats overgrowing other benthic taxa on hard substrate (Supplementary Fig. S3). This suggests that *P*. *paru* is targeting the actual cyanobacterial mat for consumption.

Our study quantified the extent to which 2 Caribbean reef fishes, not previously observed grazing benthic cyanobacterial mats, have incorporated this expanding microbial food source into their feeding behavior. Furthermore, we observed 4 other reef fishes taking bites on benthic cyanobacterial mats. Though mat-forming cyanobacteria are thought to be largely unpalatable, we present evidence that some Caribbean reef fishes are able to consume benthic cyanobacterial mats, and at high frequencies. We recommend that future work should be directed toward (1) understanding the nutritional quality of these microbial mats, (2) elucidating spatial and temporal variability in toxin production alongside spatial and temporal grazing patterns, and (3) characterizing the extent to which benthic cyanobacterial mats are being incorporated into the diets of these reef fishes. Proliferating cyanobacterial mat cover could be a key factor in causing reef baseline shifts due to their negative impact on benthic communities^17–21,23^, but might be slowed by grazing by reef fishes. Our study thus contributes to the already substantial body of literature demonstrating the importance of maintaining healthy reef fish populations to promote reef function. We argue that grazing by reef fishes may be an emerging top-down control on benthic cyanobacterial mat proliferation that warrants further study to better understand the extent that this mechanism controls mat dynamics and spatial distribution.

## Materials and Methods

### Study sites

We conducted field work in both June-July of 2018 and January of 2019 on the fringing coral reefs on the leeward side of the island of Bonaire, Netherlands, across three sites: Oil Slick Leap (N12°12.0161′ W068°18.5168′), Bachelor’s Beach (N12°07.5323′ W068°17.2388′), and Angel City (N12°06.2035′ W068°17.2328′; Supplementary Fig. S4). The only observations performed at Oil Slick Leap were of *S*. *coeruleus* during June-July of 2018. All other observations were performed at either Angel City or Bachelor’s Beach, in January 2019. At both Angel City and Bachelor’s Beach, benthic community composition was quantified within photoquadrats (n = 40 photoquadrats per site) placed at randomly determined locations along eight 10-meter transects placed parallel to the reef slope at ~10 meters depth. Photoquadrats were always placed at these randomly determined locations, and were never moved to artificially select for hard substrate (i.e. sediment was included in our sampling method). The identity of the benthos under 49 randomly allocated points in each photoquadrat was then identified to functional group (e.g., benthic cyanobacterial mat, coral, EAM, gorgonian, macroalgae, sediment, sponge) and their proportional cover estimated in the software Coral Point Count with Excel Extensions^54^ (Supplementary Fig. S5). The feeding substrate EAM has been defined as a complex and heterogenous assemblage composed of turf algae, cyanobacteria, detritus, sediment, and associated fauna^29,55^. Because these functional groups are each composed of numerous potential feeding targets of high diversity and small size, feeding observations are unable to resolve fine-scale partitioning of different feeding targets^29^. Therefore, we treat all functional groups as potential feeding substrata rather than feeding targets.

### Behavioral observations

Species observed grazing on benthic cyanobacterial mats while SCUBA diving were documented with photograph using a Nikon Coolpix W300 and/or with video using a GoPro Hero 4 equipped with a red filter. In January 2019, we conducted behavioral observations at two sites (Bachelor’s Beach and Angel City). We closely followed (1–3 meters distance) and video recorded 16 individuals of *P*. *paru* and 13 individuals of *S*. *iseri* for 11 min each (Supplementary Table S1) with a GoPro Hero 4 camera equipped with a red filter using 2.7 k video recording quality. Fish follows were performed over a period of 6 days for *P*. *paru*, and 4 days for *S*. *iseri*, between the hours of 10:00 and 16:15, close to the peak feeding time of most grazing fish species^56^ (Supplementary Table S1). When analyzing videos, each bite was recorded, and the identity of the substrate bitten was assigned to one of 8 categories: benthic cyanobacterial mat, coral, EAM, gorgonian, macroalgae, sediment, sponge, and unidentified. A bite was assigned to ‘unidentified’ when the observer’s direct line of sight of the substrate being bitten was obscured by another substrate. All protocols for this work were approved by Stichting Nationale Parken (STINAPA) Bonaire National Parks Foundation. The Florida State University internal review board (IRB) and institutional animal care and use committee (IACUC) approved our research plan, and deemed that no special permissions were required for the completion of our study because fish were followed at a distance of 1–3 meters, were not manipulated in any way, and are likely minimally influenced by diver presence from being in a popular tourist destination such as Bonaire. All methods were carried out in accordance with these approved research plans.

### Statistical analyses

Normality of the data was checked for all tests using both Shapiro-Wilk normality tests, and graphically in R (version 3.4.1). Homoscedasticity of variance was checked using both Bartlett Tests and graphically in R (version 3.4.1). Since total number of bites did not differ across sites for *P*. *paru* (ANOVA; F = 1.074, df = 1, p = 0.431) or for *S*. *iseri* (ANOVA; F = 0.269, df = 1, p = 0.614), we pooled individual follows per species (n = 16 *P*. *paru* individuals; n = 13 *S*. *iseri* individuals) across sites for statistical analyses.

We calculated binomial 95% confidence intervals for proportion of bites taken on benthic cyanobacterial mats per fish using the Clopper-Pearson Exact method with the ‘binom.confint’ function in the R (v. 3.3.2) package ‘binom’ (version1.1–1) in R (version 3.4.1; Supplementary Table S2). We used one-sample t-tests to assess if mean proportions of bites on benthic cyanobacterial mats differed significantly from mean proportions of bites taken on EAM for both species. We used Chesson’s (1978) index^57,58^ (α_i_; Supplementary Equation S1) to assess selectivity for each substrate for each species at each site. Since sponge abundance was so low and bite data on sponges was non-normally distributed, we did not calculate selectivity indices for sponges for *P*. *paru* to avoid generating biased selectivity values and interpretations. Additionally, we did not calculate selectivity indices for coral or sediment for *P*. *paru* because the number of bites taken on these substrates were so low (2 and 12 respectively) to avoid biasing selectivity indices. Wilcoxon signed rank tests were used to compare α_i_ values for each substrate to random feeding^59^ (defined as the inverse of the number of prey types available in the environment^57,58^ [*P*. *paru*, 0.25; *S*. *iseri*, 0.25]) to test preference at both sites (Supplementary Fig. S1).We also fit linear models to the number of bites and the proportion of bites on either benthic cyanobacterial mats or EAM against the total number of bites taken to better understand how bites on the major dietary components correlated with total bites taken for each *P*. *paru* individual (Supplementary Fig. S2). The data generated during this study will be deposited in the Dryad Digital Repository upon acceptance of this manuscript for publication.

## Supplementary information

Supplemental Video 1

Supplemental Video 2

Supplementary Information

## Data Availability

The data generated during this study will be deposited in the Dryad Digital Repository upon acceptance of this manuscript for publication.
