# Supplementary material for: Consumption of benthic cyanobacterial mats on a Caribbean coral reef
Source: Sci Rep. 2019 Sep 3;9:12693. doi: 10.1038/s41598-019-49126-9 (PMC6722132; doi:10.1038/s41598-019-49126-9)
Supplement: Supplementary file 3 — Supplementary Information [file 41598_2019_49126_MOESM3_ESM.pdf]

Supplementary Information for:
Consumption of benthic cyanobacterial mats on a Caribbean coral reef

Authors:
Ethan C. Cissell[1*], Joshua C. Manning[1], & Sophie J. McCoy[1]

Author Affiliation:
[1] Department of Biological Science, Florida State University, Tallahassee, Florida, USA

Corresponding Author:
*Ethan C. Cissell
319 Stadium Drive
Tallahassee, FL, 32306
ecissell@bio.fsu.edu
(850) 644-3700

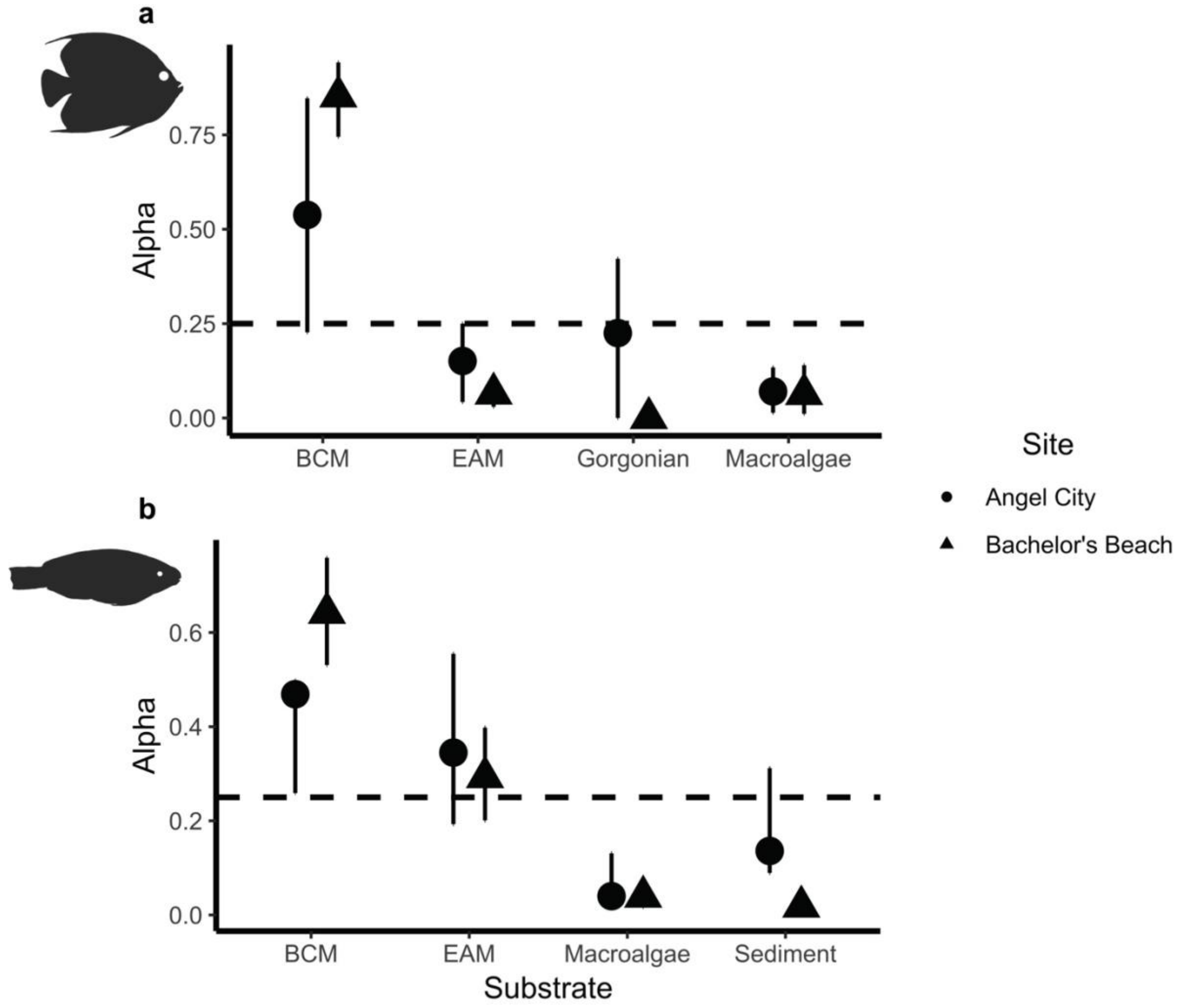

Supplemental Figure S1: We assessed selectivity for each substrate for each individual fish by calculating Chesson's (1978) indices (α_i)[1] across both sites (Angel City and Bachelor's Beach). Wilcoxon signed rank tests were then used to compare median a_i to random feeding (m^{-1})[1,2] to assess selection (significantly higher than random), avoidance (significantly lower than random), or random feeding (not significantly different from random) for each substrate for each species. Values are reported as medians (circles for Angel City and triangles for Bachelor's Beach) ± 60-95% confidence intervals (vertical lines); horizontal dashed line indicates random feeding. **a,** *Pomacanthus paru*; BCM (benthic cyanobacterial mat), Angel City: α = 0.538, p = 0.0109, v = 30, Bachelor's Beach: α = 0.852, p = 0.0156, v = 28; EAM (epilithic algal matrix), Angel City: α = 0.151, p = 0.0547, v = 4, Bachelor's Beach: α = 0.0649, p = 0.0156, v = 0; Gorgonian, Angel City: α = 0.225, p = 0.833, v = 16, Bachelor's Beach: α = 7.79E-05, p = 0.0179, v = 0; Fleshy Macroalgae, Angel City: α = 0.0703, p = 0.00781, v = 0, Bachelor's Beach: α = 0.0637, p = 0.0156, v = 0. **b,** *Scarus iseri*; BCM, Angel City: α = 0.469, p = 0.0313, v = 21, Bachelor's Beach: α = 0.642, p = 0.0156, v = 28; EAM, Angel City: α = 0.345, p = 0.0938, v = 19, Bachelor's Beach: α = 0.293, p = 0.375, v = 20; Fleshy Macroalgae, Angel City: α = 0.04, p = 0.0313, v = 0, Bachelor's Beach: α = 3.9E-02, p = 0.0156, v = 0; Sediment, Angel City: α = 0.136, p = 0.0625, v = 1, Bachelor's Beach: α = 0.0178, p = 0.0156, v = 0.

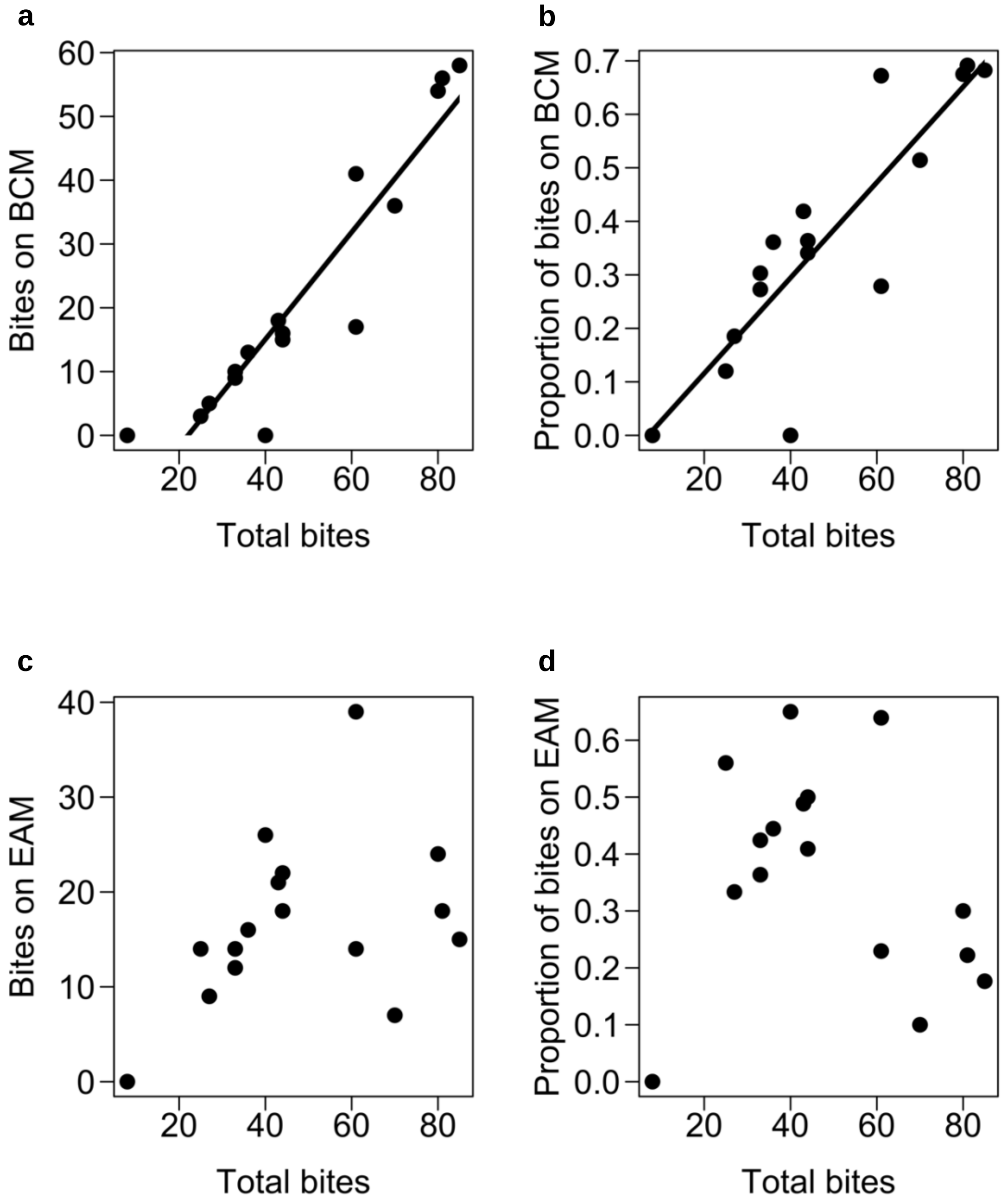

Supplemental Figure S2: To better understand how bites on the two major dietary components correlate with the total number of bites taken for each *Pomacanthus paru* individual, we fit linear models to **a,** Bites on BCM against total bites: slope estimate 0.840, SE = 0.0878, t = 9.57, DF = 14, $p < 0.001$, $R^2 = 0.858$. **b,** Proportion of bites on BCM against total bites: slope estimate 0.00892, SE = 0.00137, t = 6.54, DF = 14, $p < 0.001$, $R^2 = 0.736$. **c,** Bites on EAM against total bites taken: slope estimate 0.151, SE = 0.0967, t = 1.56, DF = 14, p = 0.142, $R^2 = 0.0869$. **d,** Proportion of bites taken on EAM against total bites: slope estimate -0.00164, SE = 0.00217, t = -0.754, DF = 14, p = 0.463, $R^2 = -0.0296$.

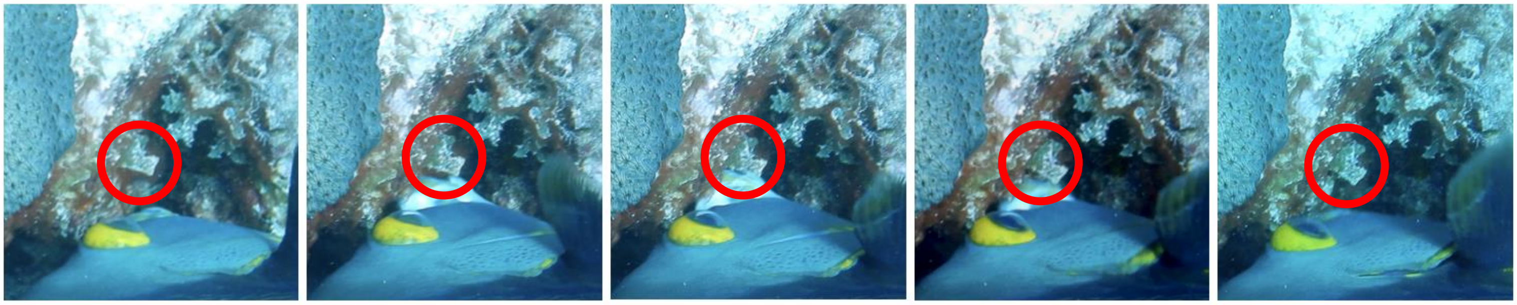

Supplemental Figure S3: Photo time-lapse of a *Pomacanthus paru* individual grazing a portion of a benthic cyanobacterial mat (tracked with a red circle) overgrowing *Dictyota* spp. growing on hard substrate, while largely avoiding consumption of the underlying *Dictyota* spp.

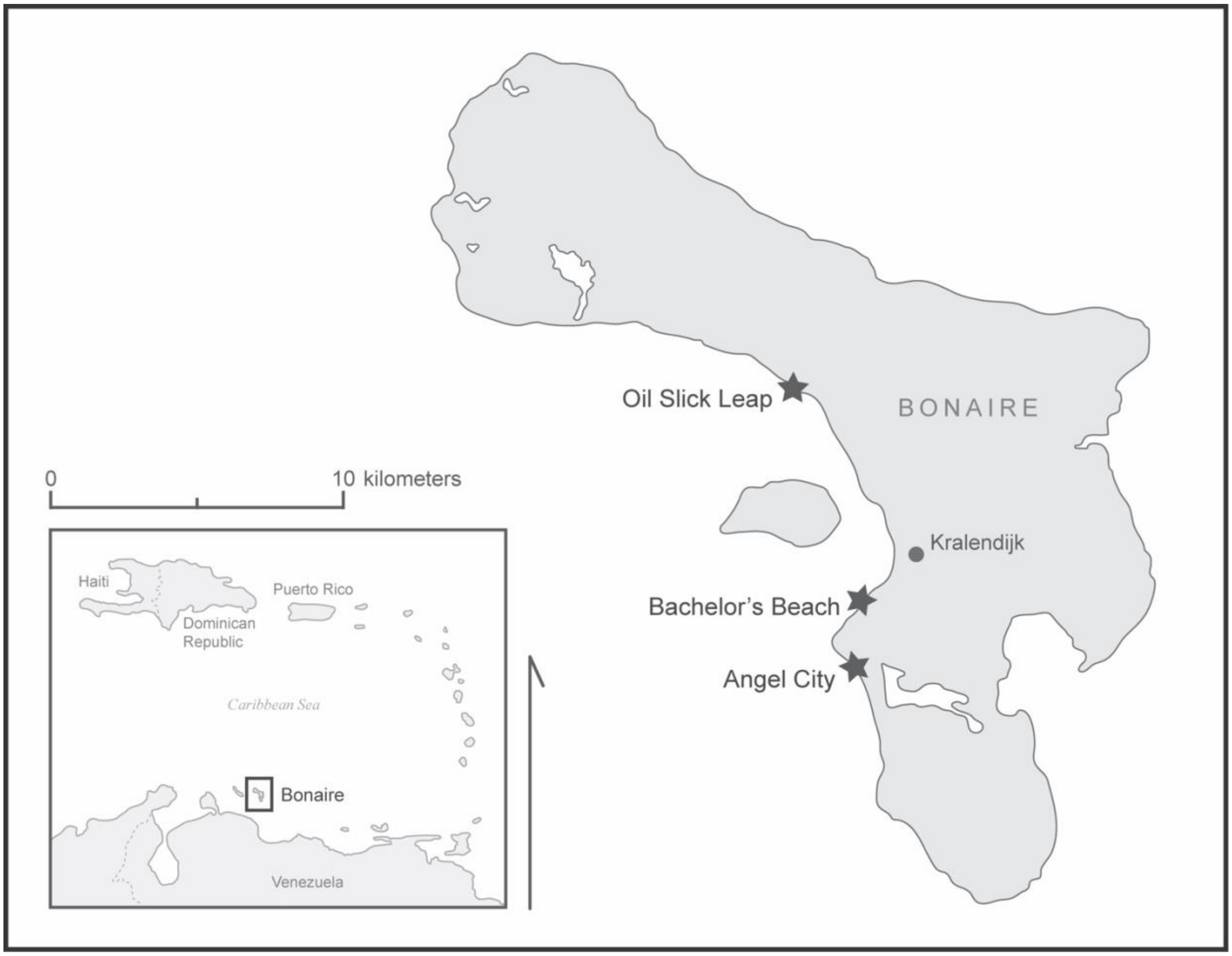

Supplemental Figure S4: Location of study sites (marked with stars) on the island of Bonaire, Netherlands. June-July 2018 observations were conducted exclusively at the northern site, Oil Slick Leap. January 2019 observations were conducted exclusively at the two southern sites: Bachelor's Beach and Angel City. *Inset*: Location of Bonaire (marked with box) in the southern Caribbean Sea. Map was made by J.R. Cissell.

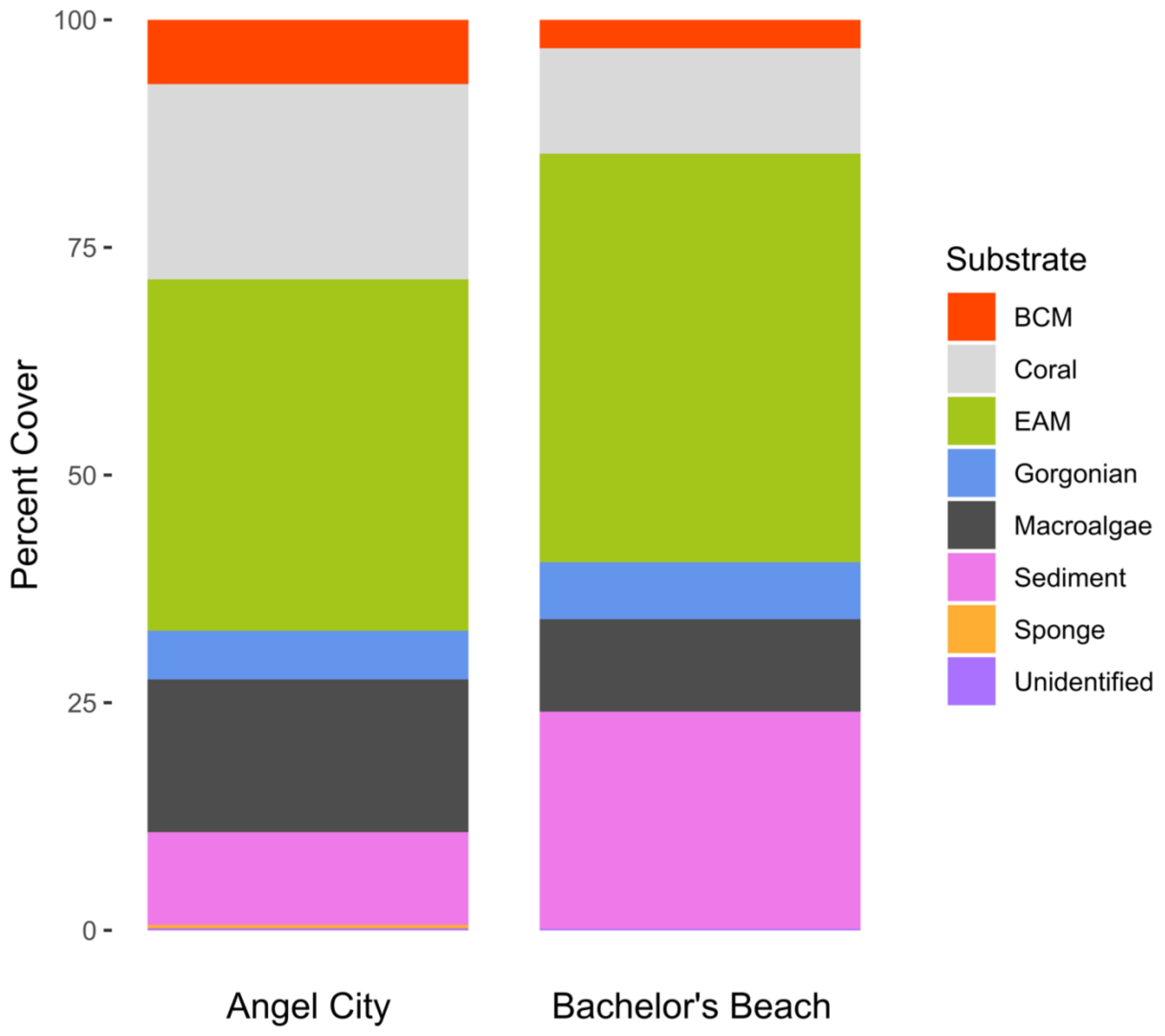

Supplemental Figure S5: Stacked bar charts showing percent cover of the bitten substrata at Angel City and Bachelor's Beach assessed at n=40 photoquadrats across 8, 10m transects per site. **Angel City**: BCM = 7.07% ± 3.50 (mean ± 95% confidence interval); Coral = 21.4% ± 5.76; EAM = 38.6% ± 7.91; Gorgonian = 5.35% ± 2.22; Fleshy Macroalgae (largely *Dictyota* spp.) = 16.8% ± 4.95; Sediment = 10.2% ± 5.1; Sponge = 0.371% ± 0.619; Unidentified = 0.219% ± 0.234. **Bachelor's Beach**: BCM = 3.11% ± 2.11 (mean ± 95% confidence interval); Coral = 11.6% ± 4.09; EAM = 44.9% ± 8.3; Gorgonian = 6.27% ± 4.4; Fleshy Macroalgae = 10.2% ± 3.55; Sediment = 23.9% ± 6.02; Sponge = 0.00%; Unidentified = 0.161% ± 0.219.

Supplemental Table S1: All individual follows listed by species with the location (Site), date of follow (Date), start time of the follow on a 24hr clock (Start), fish identification number (ID), length of the follow in seconds (Duration), total number of bites taken (Total Bites), and the number of bites per minute (Bite Rate). Follows had a mean duration of 662 sec ± 49.9 sec standard deviation.

Species	Site	Date	Start	ID	Duration	Total Bites	Bite Rate
P. paru	Angel City	1/16/19	11:38	1	672	40	3.57
		1/16/19	11:50	2	633	81	7.68
		1/16/19	13:43	3	704	70	5.97
		1/19/19	16:00	4	697	61	5.25
		1/19/19	16:02	5	614	43	4.21
		1/19/19	11:12	6	601	85	8.49
		1/22/19	—	7	676	27	2.40
		1/24/19	11:45	8	690	25	2.17
	Bachelor's Beach	1/18/19	16:02	9	792	33	2.50
		1/18/19	11:30	10	644	33	3.07
		1/18/19	11:46	11	610	36	3.54
		1/18/19	13:45	12	657	44	4.02
		1/18/19	14:21	13	637	61	5.75
		1/23/19	10:31	14	700	80	6.86
		1/23/19	12:37	15	696	8	0.69
		1/23/19	13:05	16	730	44	3.62
S. iseri	Angel City	1/19/19	13:04	1	653	187	17.2
		1/19/19	13:54	2	572	250	26.2
		1/19/19	15:19	3	641	317	29.7
		1/19/19	15:31	4	608	182	18.0
		1/24/19	11:57	5	684	201	17.6
		1/24/19	12:23	6	733	227	18.6
	Bachelor's Beach	1/20/19	15:29	7	746	285	22.9
		1/23/19	10:05	8	606	235	23.3
		1/23/19	10:43	9	666	251	22.6
		1/23/19	12:24	10	637	205	19.3
		1/23/19	12:50	11	622	208	20.1
		1/23/19	14:47	12	626	288	27.6
		1/23/19	15:00	13	644	207	19.3

Supplemental Table S2: Proportion of bites on benthic cyanobacterial mats (Prop) with lower and upper bounds of binomial 95% confidence intervals (Lower; Upper, respectively) calculated using the Clopper-Pearson Exact method for each fish. Also given are the total number of bites taken on all substrates (Total Bites), and the number of bites taken on benthic cyanobacteria (BCM Bites). For *Pomacanthus paru*, 95% confidence intervals on proportion of bites on BCM only included 0 on 2 out of 16 fish observed (2 individuals took 0 bites on BCM). For *Scarus iseri*, 95% confidence intervals never included 0 for proportion of bites on BCM.

Species	ID	Total Bites	BCM Bites	Prop	Lower	Upper
P. paru	1	40	0	0	0	0.0881
	2	81	56	0.691	0.579	0.789
	3	70	36	0.514	0.392	0.636
	4	61	17	0.279	0.171	0.408
	5	43	18	0.419	0.27	0.579
	6	85	58	0.682	0.572	0.779
	7	27	5	0.185	0.063	0.381
	8	25	3	0.12	0.0255	0.312
	9	33	10	0.303	0.156	0.487
	10	33	9	0.273	0.133	0.455
	11	36	13	0.361	0.208	0.538
	12	44	16	0.364	0.224	0.522
	13	61	41	0.672	0.54	0.787
	14	80	54	0.675	0.561	0.776
	15	8	0	0	0	0.369
	16	187	32	0.171	0.12	0.233
S. iseri	1	250	42	0.168	0.124	0.22
	2	317	55	0.174	0.133	0.22
	3	182	38	0.209	0.152	0.275
	4	201	46	0.229	0.173	0.293
	5	227	16	0.0705	0.0408	0.112
	6	285	55	0.193	0.149	0.244
	7	235	20	0.0851	0.0528	0.128
	8	235	20	0.0851	0.0528	0.128
	9	251	19	0.0757	0.0462	0.116
	10	205	43	0.21	0.156	0.272
	11	208	18	0.0865	0.0521	0.133
	12	288	40	0.139	0.101	0.184
	13	207	29	0.14	0.0959	0.195

Supplemental Table S3: Number of feeding bouts exceeding 5 consecutive bites on the same substrate (Bouts) listed per substrate for both focal species.

Species	Substrate	Bouts
P. paru	BCM	18
	Coral	0
	EAM	14
	Gorgonian	2
	Macroalgae	0
	Sediment	0
	Sponge	1
	Unidentified	1
S. iseri	BCM	20
	Coral	0
	EAM	122
	Gorgonian	0
	Macroalgae	2
	Sediment	7
	Sponge	0
	Unidentified	0

$$\boldsymbol{\alpha_i = \frac{r_i/p_i}{\sum_{j=1}^{m} r_j/p_j} \quad , \quad i = 1, \ldots, m}$$

Supplemental Equation S1: Equation used to calculate selectivity indices where α_i is the selectivity for substrate type i, r_i is the relative abundance of substrate type i in the diet, p_i is the relative abundance of substrate type i in the environment, and m is the number of different substrate types available in the environment.

Supplemental Video S1: Video showing a portion of a fish follow conducted on a *Pomacanthus paru* individual demonstrating prolonged feeding on a single benthic cyanobacterial mat.

Supplemental Video S2: Video showing a portion of a fish follow conducted on a *Scarus iseri* individual demonstrating roving feeding on different benthic cyanobacterial mats.

Supplemental References:

1. Chesson, J. Measuring preference in selective predation. *Ecology* **59,** 211-215 (1978).
2. Juanes, F., Buckel, J. & Scharf, F. Predatory behaviour and selectivity of a primary piscivore: comparison of fish and non-fish prey. *Mar. Ecol. Prog. Ser.* **217,** 157–165 (2001).
